# The intercropping partner affects arbuscular mycorrhizal fungi and *Fusarium oxysporum* f. sp. *lycopersici* interactions in tomato

**DOI:** 10.1007/s00572-013-0495-x

**Published:** 2013-04-03

**Authors:** Karin Hage-Ahmed, Johannes Krammer, Siegrid Steinkellner

**Affiliations:** Department of Crop Sciences, Division of Plant Protection, University of Natural Resources and Life Sciences, Vienna, Peter-Jordan-Strasse 82, 1190 Vienna, Austria

**Keywords:** AM fungi, *Fusarium oxysporum* f. sp*. lycopersici*, Intercropping, *Solanum lycopersicum*, Biological control

## Abstract

Arbuscular mycorrhizal fungi (AMF) and their bioprotective aspects are of great interest in the context of sustainable agriculture. Combining the benefits of AMF with the utilisation of plant species diversity shows great promise for the management of plant diseases in environmentally compatible agriculture. In the present study, AMF were tested against *Fusarium oxysporum* f. sp. *lycopersici* with tomato intercropped with either leek, cucumber, basil, fennel or tomato itself. Arbuscular mycorrhizal (AM) root colonisation of tomato was clearly affected by its intercropping partners. Tomato intercropped with leek showed even a 20 % higher AM colonisation rate than tomato intercropped with tomato. Positive effects of AMF expressed as an increase of tomato biomass compared to the untreated control treatment could be observed in root as well as in shoot weights. A compensation of negative effects of *F. oxysporum* f. sp. *lycopersici* on tomato biomass by AMF was observed in the tomato/leek combination. The intercropping partners leek, cucumber, basil and tomato had no effect on *F. oxysporum* f. sp. *lycopersici* disease incidence or disease severity indicating no allelopathic suppression; however, tomato co-cultivated with tomato clearly showed a negative effect on one plant/pot with regard to biomass and disease severity of *F. oxysporum* f. sp. *lycopersici*. Nonetheless, bioprotective effects of AMF resulting in the decrease of *F. oxysporum* f. sp. *lycopersici* disease severity were evident in treatments with AMF and *F. oxysporum* f. sp. *lycopersici* co-inoculation. However, these bioprotective effects depended on the intercropping partner since these effects were only observed in the tomato/leek and tomato/basil combination and for the better developed plant of tomato/tomato. In conclusion, the effects of the intercropping partner on AMF colonisation of tomato are of great interest for crop plant communities and for the influences on each other. The outcome of the bioprotective effects of AMF resulting in the decrease on *F. oxysporum* f. sp. *lycopersici* disease severity and/or compensation of plant biomass does not depend on the degree of AM colonisation but more on the intercropping partner.

## Introduction

Arbuscular mycorrhizal fungi (AMF) are the most prevalent type of mycorrhizal fungi and form a mycorrhizal symbiosis with a wide range of vascular plants including many important crop species (Smith and Read 2008). Apart from improved plant nutrition, AMF are reputed to control a number of plant diseases, especially soil-borne diseases (Azcón-Aguilar et al. 2002; Whipps 2004; Singh et al. 2000; Xavier and Boyetchko 2004; St-Arnaud and Vujanovic 2007; Newsham et al. 1995). This is of high significance in the field of sustainable agriculture, where the input of fertilisers and chemical plant protectants is reduced or even absent. Furthermore, it is known that AMF have an impact on plant community structure and diversity by altering inter- or intraspecific competitive situations (Smith and Read 2008; van der Heijden et al. 2003; Schroeder-Moreno and Janos 2008). In return, however, arbuscular mycorrhizal (AM) fungal community structure can be influenced by the host plants (Smith and Read 2008; Bever 2002). These effects are well described for grassland communities; for crop species, only Schroeder-Moreno and Janos (2008) have reported similar effects. For the application of intercropping in combination with AMF, these feedback matters need to be kept in mind and need to be tested for each intercropping arrangement separately. Thus, further research is necessary for the application to crop species, especially with regard to intercropping arrangements with the aim of improved plant performance, which also implies lower infection rates caused by diseases. Plant species diversity could make a significant contribution to the reduction of plant diseases (Ratnadass et al. 2012). This would reduce the use of chemical pesticides and therefore reduce adverse effects on humans and the environment.

*Fusarium oxysporum* f. sp. *lycopersici* is a soil-borne fungus, which invades the plants through the roots and causes wilting in tomato which can result in severe yield losses. Apart from the environmental issues mentioned before, chemical control of soil-borne pathogens is difficult to impossible, thus, giving a further strong reason for alternative methods of disease control.

It has been shown previously that AMF reduced adverse effects of *F. oxysporum* f. sp. *lycopersici* in tomato when co-inoculated with this pathogen (Dehne and Schönbeck 1979; Akköprü and Demir 2005). Apart from improved plant nutrition mechanisms like changes in root and vessel system, mycorrhizosphere effects and induced systemic resistance are involved in these effects (Akköprü and Demir 2005; Dehne and Schönbeck 1979; Whipps 2004). However, this bioprotective effect depends on the AM fungal identity (Sikes et al. 2009), making it crucial to choose the proper AMF isolates. Furthermore, it has been shown that allelopathic suppression by root exudates can occur in intercropping arrangements, like for tomato and Chinese chive against *Pseudomonas solanacearum* (Yu 1999) and for watermelon and rice against *Fusarium oxysporum* f. sp. *niveum* (Hao et al. 2010)*.* A combination of the bioprotective effects of AMF and intercropping partners can be considered as a new potential strategy against soil-borne pathogens and would be of high significance for sustainable agriculture.

In our work, we focused on tomato (*Solanum lycopersicum* L.) intercropped with leek (*Allium porrum* L.) and basil (*Ocimum basilicum* L.), commonly known as stimulating species, and fennel (*Foeniculum vulgare* (L.) Mill.) and cucumber (*Cucumis sativus* L.), known as species with adverse effects on tomato, in combination with a commercially available AMF inoculum. We hypothesised that the intercropping partners of tomato can have conducive, adverse or neutral effects on AMF and tomato wilt caused by *F. oxysporum* f. sp. *lycopersici*. The aim was to investigate (1) the AMF colonisation rate of tomato in intercropping settings, (2) the influence of AMF application in intercropping systems on *F. oxysporum* f. sp. *lycopersici* disease severity and (3) the effects of AMF and/or *F. oxysporum* f. sp. *lycopersici* in different intercropping systems on root and shoot weights of tomato.

## Material and methods

### Plant and fungal material

Tomato (*S. lycopersicum* L. cv. Kremser Perle), leek (*A. porrum* L. cv. Golem), fennel (*F. vulgare* (L.) Mill. cv. Fino), basil (*O. basilicum* L. cv. Genovese) and cucumber (*C. sativus* L. cv. Aztec F1) were used as crop plants. All seeds were surface-sterilised by soaking in 50 % household bleach (‘Dan Klorix’, 3.8 % NaOCl) for 10 min and rinsed afterwards three times with autoclaved distilled water. Seeds were transferred to pots filled with autoclaved perlite (Granuperl S 3–6, Knauf Perlite GmbH, Vienna, Austria) and incubated in a growth chamber (York International) with a 16-h light (light intensity 296 μmol m^−1^ s^−1^) and 8-h dark photoperiod at 24 °C. The perlite was irrigated with tap water. Seeds of leek and fennel were pre-cultivated 6 weeks, and seeds of tomato and basil 3 weeks, before transplanting. Due to the rapid growth, cucumber seeds were used in the below described plant bioassay without pre-cultivation.

*F. oxysporum* f. sp. *lycopersici* (*F. oxysporum* f. sp. *lycopersici*007) was cultivated for 2 weeks at 24 °C in darkness on Czapek Dox Agar (Duchefa Biochemie, Haarlem, The Netherlands). For plant inoculation, a microconidial suspension was prepared by flooding the *F. oxysporum* f. sp. *lycopersici* colonies with sterile, distilled water and gently rubbing with a Drigalski spatula. Thereafter, the conidial suspension was filtered through three layers of cheese cloth (fleece filters, 20–150 μm pore diameter, Laporte, Wels, Austria) and adjusted to a final concentration of 10^5^ microconidia ml^−1^.

For AMF plant inoculation, a commercially available inoculum (Symbivit®, Zivojin Rilakovic, Guntramsdorf, Austria) was used. This inoculum contains at least 80,000 spores l^−1^ and comprises six *Glomus* species (*Glomus etunicatum*, *Glomus microagregatum*, *Glomus intraradices*, *Glomus claroidium*, *Glomus mosseae* and *Glomus geosporum*).

### Plant bioassay

Pre-cultivated plantlets of tomato, basil, leek and fennel and cucumber seeds were transferred to pots (volume 1,183 cm^−3^) filled with an autoclaved (20 min at 121 °C) mixture of sand, soil and expanded clay (1:1:1, *v*/*v*/*v*). The plants were cultivated as a dual culture system with the following plant combinations: tomato/tomato, tomato/basil, tomato/cucumber, tomato/fennel and tomato/leek. The experimental set-up included four different treatments for each dual culture: (1) *F. oxysporum* f. sp. *lycopersici*, (2) AMF, (3) *F. oxysporum* f. sp. *lycopersici* and AMF and (4) a control without *F. oxysporum* f. sp. *lycopersici* and without AMF. For each treatment three replicates comprising 6 pots each were used, giving 72 pots per plant combination and a total of 360 pots.

For the AMF treatment, 4 ml of the AMF inoculum were added to the planting hole at the potting procedure. *F. oxysporum* f. sp. *lycopersici* was applied to the plant roots by dipping the roots for 5 min in a microconidial suspension (10^5^ microconidia ml^−1^) before plants were transferred to the pots. For the AMF + *F. oxysporum* f. sp. *lycopersici* treatment, both inocula were added as mentioned above.

The plants were grown in a random design in a greenhouse for 11 weeks and were irrigated according to their moisture requirements with a nutrient solution (Steinkellner et al. 2005).

After 11 weeks, the plants were gently removed from the substrate und washed thoroughly under tap water. Root and shoot fresh weights were determined. Plants of the tomato/tomato combination were separated into two groups per pot according to their root weights to assess intraspecific effects.

Disease incidence was calculated according to the following formula:

$$ \mathrm{Disease}\ \mathrm{incidence}=\frac{\mathrm{Number}\ \mathrm{of}\ \mathrm{infected}\ \mathrm{plants}}{\mathrm{Total}\ \mathrm{number}\ \mathrm{of}\ \mathrm{plants}}\times 100 $$

*F. oxysporum* f. sp. *lycopersici* disease severity was determined by measuring the amount of vessel discolouration of the stem in relation to the total stem length (length of infected stem [cm]/total length of stem [cm]). Leaf symptoms were not evident at this plant stage and were therefore not considered for disease severity assessment. For confirmation of *F. oxysporum* f. sp. *lycopersici* infection, segments of 2-cm length starting upwards the shoot basis were dipped in 70 % ethanol, flamed and put into Petri dishes containing potato dextrose agar amended with antibiotics to prevent bacterial growth according to Steinkellner et al. (2011). The determination of *F. oxysporum* f. sp. *lycopersici* was done according to Nelson et al. (1983) by visual and microscopic analyses.

Defined root segments of 1-cm length, starting 2 cm down the shoot, were used for determining the degree of mycorrhization. The root segments were cleared by boiling for 4 min (tomato, cucumber, leek and fennel) and 5 min (basil), respectively, in 10 % KOH and afterwards rinsed three times with tap water. Roots were stained by boiling for 3 min in a 5 % ink–vinegar solution (Vierheilig et al. 1998). The percentage of root colonisation was determined according to the method of McGonigle et al. (1990).

### Statistical analyses

AM colonisation rate was rank-transformed and analysed by using one-way ANOVA and Bonferroni’s test. Data of disease severity were analysed by Kruskal–Wallis and Mann–Whitney *U* test. Since neither root nor shoot weights met the homogeneity assumption of variance, even after transformation, a two-way ANOVA could not be applied. Therefore, data were analysed by one-way ANOVA (cross-checked with Welch’s ANOVA) and Tamhane’s test. Correlation analyses were based on Spearman’s rho. All statistical analyses were performed using PASW Statistics 18.

## Results

### AM colonisation

**Fig. 1:**
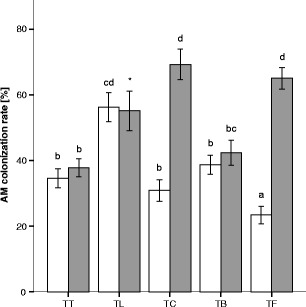
AM colonisation rate (%) of different plant species in different intercropping combinations (mean ± standard error). *T* = tomato, *L* = leek, *C* = cucumber, *B* = basil, *F* = fennel; *empty bars* represent the tomato plants and *grey bars* the corresponding intercropping partner. Plants were only inoculated with AMF. *Different letters* indicate significant difference according to ANOVA and Bonferroni’s test (*P* < 0.05). * excluded from statistical analysis

The intercropping partner significantly influenced the AM colonisation levels of tomato plants (*F*_8,151_ = 18.761, *P* < 0.0001) (Fig. 1). Tomato plants intercropped with leek showed a 20 % higher colonisation level of the roots than tomato co-cultivated with tomato, whereas, tomato intercropped with fennel showed a 13 % lower AM colonisation level. Tomato intercropped with cucumber and basil, respectively, did not reveal any differences in the AM colonisation level compared to the tomato/tomato (TT) combination. Within the TT combination, tomato plants grown in one pot did not show any differences in their AM colonisation levels. Tomato and basil plants in the tomato/basil (TB) combination did not show any significant differences in their AM colonisation rates, whereas for tomato and cucumber in the tomato/cucumber (TC) combination, cucumber had a 2.3-fold higher AM colonisation level than tomato. Furthermore, fennel in the tomato/fennel (TF) combination had a 2.7-fold higher colonisation level than the tomato plants. The AM colonisation level of leek (55.11 ± 6.0) in the tomato/leek (TL) combination was not considered for ANOVA due to its high variance caused by the small root system of the leek plants. However, AM colonisation level was similar to the one of tomato in the TL combination. AM colonisation levels are only shown for the AMF treatment since *F. oxysporum* f. sp. *lycopersici* did not show an influence on the AM colonisation levels compared to the AMF treatment alone. Plants of the control and *F. oxysporum* f. sp. *lycopersici* treatment were also checked for AM colonisation but did not show any presence of AMF.

### Assessment of *F. oxysporum* f. sp. *lycopersici* disease incidence and disease severity

*F. oxysporum* f. sp. *lycopersici* disease incidence and disease severity are presented in Table 1. For the TT combination, means of all plants from pots are given. Also, tomato plants of one pot were separated according to their root weights (see also Table 2) to see intraspecific effects and presented as Tomato_1 (T_1) and Tomato_2 (T_2). When considering all plants, the disease incidence of TT was 80.6 and 52.9 % in the *F. oxysporum* f. sp. *lycopersici* and AMF + *F. oxysporum* f. sp. *lycopersici* treatments, respectively, so that disease incidence was 35 % less in the AMF + *F. oxysporum* f. sp. *lycopersici* treatment than in the *F. oxysporum* f. sp. *lycopersici* treatment. In the other dual cultures, AMF + *F. oxysporum* f. sp. *lycopersici* also showed lower disease incidences than the *F. oxysporum* f. sp. *lycopersici* treatment alone. The plant combinations had no impact on *F. oxysporum* f. sp. *lycopersici* disease incidence. With regards to root weights of the tomato plants of one pot, disease incidence in the TT combination of T_1 was reduced in the AMF + *F. oxysporum* f. sp. *lycopersici* treatment (37.5 %) and, consequently, showed almost 50 % less disease incidence than in the *F. oxysporum* f. sp. *lycopersici* treatment (72.2 %).

**Table 1:** *F. oxysporum* f. sp. *lycopersici* disease incidence and disease severity (%) in the different plant combinations (mean ± S.E.)

Dual culture	Disease incidence (%)		Disease severity (%)	
	*F. oxysporum* f. sp. *lycopersici*	AMF + *F. oxysporum* f. sp. *lycopersici*	*F. oxysporum* f. sp. *lycopersici*	AMF + *F. oxysporum* f. sp. *lycopersici*
Tomato/tomato^a^	80.6	52.9	16.2 ± 2.1 A a	14.32 ± 3.8 A a
Tomato/leek	66.7	44.4	14.3 ± 3.4 B a	4.4 ± 1.5 A a
Tomato/cucumber	81.3	55.6	15.8 ± 3.9 A a	7.0 ± 2.2 A a
Tomato/basil	72.2	55.6	14.2 ± 2.9 B a	5.3 ± 1.7 A a
Tomato/fennel	66.7	55.6	14.0 ± 2.8 A a	6.9 ± 2.0 A a
Tomato/tomato (T_1)^b^	72.2	37.5	11.4 ± 2.4 B x	3.8 ± 1.3 A x
Tomato/tomato (T_2)^c^	88.8	66.7	21.0 ± 3.0 A y	23.7 ± 6.4 A y

Different letters indicate significant differences, small letters among columns (Kruskal–Wallis test, *P* < 0.05), capital letters among rows (Mann–Whitney *U* test, *P* < 0.05).

^a^Data were calculated for both plants/pot

^b^Data were calculated for the stronger tomato plant/pot according to the root weight

^c^Data were calculated for the weaker tomato plant/pot according to the root weight

**Table 2:** Root weights (in grams) of tomato plants grown in a dual culture system (mean ± S.E.)

Dual culture	Treatment				
	Control	*F. oxysporum* f. sp. *lycopersici*	AMF	AMF + *F. oxysporum* f. sp. *lycopersici*	
Tomato/tomato (T_1)^a^	6.36 ± 0.44 (A) cd	5.76 ± 0.41 (A) c	7.11 ± 0.48 (B) bc	4.90 ± 0.36 (A) bc	*F*_(3,66)_ = 4.549
					*P* < 0.01
Tomato/tomato (T_2)^b^	3.71 ± 0.24 (B) a	2.42 ± 0.26 (A) a	4.42 ± 0.39 (B) a	2.45 ± 0.35 (A) a	*F*_(3,67)_ = 9.713
					*P* < 0.0001
Tomato/leek	7.03 ± 0.22 (A) d	5.87 ± 0.46 (A) c	8.47 ± 0.46 (B) d	6.06 ± 0.39 (AB) c	*F*_(3,67)_ = 8.947
					*P* < 0.0001
Tomato/ cucumber	5.14 ± 0.46 (B) abc	2.70 ± 0.35 (A) ab	5.42 ± 0.54 (B) ab	3.55 ± 0.47 (AB) ab	*F*_(3,66)_ = 7.095
					*P* < 0.0001
Tomato/basil	7.15 ± 0.36 (B) d	5.57 ± 0.40 (A) c	8.90 ± 0.27 (B) d	6.79 ± 0.73 (AB) c	*F*_(3,68)_ = 8.424
					*P* < 0.0001
Tomato/fennel	4.80 ± 0.15 (A) b	4.32 ± 0.41 (A) bc	6.23 ± 0.26 (B) b	5.42 ± 0.22 (AB) c	*F*_(3,68)_ = 9.006
					*P* < 0.0001
	*F*_(5,101)_ = 16.695	*F*_(5,100)_ = 15.735	*F*_(5,101)_ = 17.336	*F*_(5,101)_ = 12.823	
	*P* < 0.0001	*P* < 0.0001	*P* < 0.0001	*P* < 0.0001	

Different letters indicate significant differences; capital letters among rows, i.e. plant combinations and letters among columns, i.e. treatments (ANOVA and Tamhane’s test *P* < 0.05)

^a^Data were calculated for the stronger tomato plant/pot according to the root weight

^b^Data were calculated for the weaker tomato plant/pot according to the root weight

Disease severity within the *F. oxysporum* f. sp. *lycopersici* treatment ranged between 14.0 and 16.2 % and between 4.4 and 14.32 % within the AMF + *F. oxysporum* f. sp. *lycopersici* treatment. The plant combinations had neither in the *F. oxysporum* f. sp. *lycopersici* (*χ* ^2^_(4)_ = 0.412, *P* = 0.981) nor in the AMF + *F. oxysporum* f. sp. *lycopersici* treatment (*χ* ^2^_(4)_ = 0.195, *P* = 0.700) a significant influence on *F. oxysporum* f. sp. *lycopersici* disease severity. Within the TL and TB combinations, AMF + *F. oxysporum* f. sp. *lycopersici* reduced disease severity significantly by 70 % (*P* < 0.05) and 63 % (*P* < 0.05), respectively. In the other plant combinations, the AMF + *F. oxysporum* f. sp. *lycopersici* treatment also tended to show lower disease severity. Within T_1, *F. oxysporum* f. sp. *lycopersici* showed higher disease severity than AMF + *F. oxysporum* f. sp. *lycopersici* (*P* < 0.05). Within T_2, disease severity was similar between *F. oxysporum* f. sp. *lycopersici* and AMF + *F. oxysporum* f. sp. *lycopersici* (*P* = 0.703). Within the *F. oxysporum* f. sp. *lycopersici* (*P* < 0.05) as well as in the AMF + *F. oxysporum* f. sp. *lycopersici* (*P* < 0.05) column, T_1 had significantly lower disease severity than T_2. There was no significant correlation between disease severity and AM colonisation rate (*P* = 0.778, *R* ^*2*^ = 0.0008).

### Effect of intercropping, AMF and *F. oxysporum* f. sp. *lycopersici* on tomato growth

Plant growth of tomato in the different plant combinations and treatments was assessed by root and shoot weights.

#### Root effects

Root weights for the treatments with *F. oxysporum* f. sp. *lycopersici* and/or AMF application within the different plant combinations are shown in Table 2. The factor ‘intercropping partner’ had in each treatment a significant effect on tomato root weights (for *p* values, see Table 2). Root weights of T_1 and T_2 of the TT combination in the control treatment differed significantly, indicating an intraspecific effect of tomato plants. Plants of T_2 had almost 50 % less weight than the ones from T_1. TL and TB showed the highest tomato root weights, whilst TC and TF ranged between the lowest and the highest root weights. These trends were also seen in the *F. oxysporum* f. sp. *lycopersici*, AMF and AMF + *F. oxysporum* f. sp. *lycopersici* treatments.

The factor ‘treatment’ had in each dual culture a significant influence on root weights (for *p* values, see Table 2). For T_2 within the TT treatment, *F. oxysporum* f. sp. *lycopersici* as well as AMF + *F. oxysporum* f. sp. *lycopersici* significantly reduced the root weight compared to the control treatment. For T_1, this effect could not be observed. A significant reduction of the root weights in the *F. oxysporum* f. sp. *lycopersici* treatment compared to the control could also be observed in TC. The AMF treatment over all plant combinations showed similar root weights as the corresponding control treatments, apart from TL, TF and T_1 of TT which showed an increase in root weights. AMF + *F. oxysporum* f. sp. *lycopersici* over all plant combinations did not show a change in root weights compared to the *F. oxysporum* f. sp. *lycopersici* or control treatment, apart from AMF + *F. oxysporum* f. sp. *lycopersici* of T_2, where the root weight was reduced compared to the control. There was no significant correlation between root weight and AM colonisation rate (*P* = 0.113, *R* ^*2*^ = 0.012); however, root weight was negatively correlated with disease severity (*P* < 0.0001, *R* ^*2*^ = 0.233).

#### Shoot effects

Shoot weights of tomato plants from the different plant combinations are shown in Table 3. The factor ‘intercropping partner’ had a significant effect on tomato shoot weights in each treatment (for *p* values see Table 3). Within the control treatment, T_2 of the TT combination showed around 40 % less shoot weight than T_1. The highest shoot weights were reached by tomato plants of the TB and TL combinations (increases up to 320 and 180 %, compared to T_2 and T_1, respectively). The shoot weights of the tomato plants of the TC and TF combinations ranged between 11.49 and 13.06 g and were therefore higher than the shoot weights of T_2 (6.23 g).

**Table 3:** Shoot weights (in grams) of tomato plants grown in a dual culture system (mean ± S.E.)

Dual culture	Treatment				
	Control	*F. oxysporum* f. sp. *lycopersici*	AMF	AMF + *F. oxysporum* f. sp. *lycopersici*	
Tomato/tomato (T_1)^a^	10.79 ± 0.83 (A) b	10.11 ± 0.70 (A) bc	19.17 ± 1.32 (B) b	16.66 ± 1.60 (B) bc	*F*_(3,66)_ = 15.279
					*P* < 0.0001
Tomato/tomato (T_2)^b^	6.23 ± 0.53 (B) a	4.15 ± 0.28 (A) a	10.22 ± 1.08 (C) a	8.21 ± 1.49 (ABC) a	*F*_(3,67)_ = 7.398
					*P* < 0.0001
Tomato/leek	18.67 ± 0.72 (B) c	14.10 ± 1.20 (A) cd	31.73 ± 1.31 (C) d	28.71 ± 2.32 (C) d	*F*_(3,67)_ = 30.005
					*P* < 0.0001
Tomato/ cucumber	11.49 ± 0.66 (A) b	7.95 ± 1.22 (A) ab	16.72 ± 1.50 (B) b	12.62 ± 1.94 (AB) ab	*F*_(3,66)_ = 6.293
					*P* = 0.001
Tomato/basil	19.05 ± 0.86 (A) c	16.85 ± 1.21 (A) d	27.94 ± 1.96 (B) d	21.77 ± 2.11 (AB) cd	*F*_(3,68)_ = 8.789
					*P* < 0.0001
Tomato/fennel	13.06 ± 0.58 (A) b	11.15 ± 0.80 (A) bc	23.78 ± 1.67 (B) bc	21.96 ± 1.77 (B) c	*F*_(3,66)_ = 23.058
					*P* < 0.0001
	*F*_(5,101)_ = 48.299	*F*_(5,100)_ = 22.359	*F*_(5,101)_ = 26.616	*F*_(5,100)_ = 15.169	
	*P* < 0.0001	*P* < 0.0001	*P* < 0.0001	*P* < 0.0001	

Different letters indicate significant differences; capital letters among rows, i.e. plant combinations and letters among columns, i.e. treatments (ANOVA and Tamhane’s test *P* < 0.05)

^a^Data were calculated for the stronger tomato plant/pot according to the root weight

^b^Data were calculated for the weaker tomato plant/pot according to the root weight

The factor ‘treatment’ had a significant influence on shoot weights in each dual culture (for *p* values, see Table 3). In the TT combination, shoot weights of T_1 showed a significant increase of 70–80 % in the AMF and AMF + *F. oxysporum* f. sp. *lycopersici* treatments, compared to the control and *F. oxysporum* f. sp. *lycopersici* treatment. A similar picture can be seen within the TL and TF combinations, where the shoot weights increased up to 200–230 % and 170–210 %, respectively, compared to the control and *F. oxysporum* f. sp. *lycopersici* treatment. In T_2 of the TT combination, the *F. oxysporum* f. sp. *lycopersici* treatment reduced the shoot weights of the plants compared to the control and the AMF treatment. On the other hand, AMF as well as AMF + *F. oxysporum* f. sp. *lycopersici* increased the shoot weights as compared to the control and the *F. oxysporum* f. sp. *lycopersici* treatment in T_1 of the TT combination, the TL and the TF combinations. In T_2 of the TT combination, the TC and TB combinations, AMF increased the shoot weights compared to the *F. oxysporum* f. sp. *lycopersici* treatment, whereas the AMF + *F. oxysporum* f. sp. *lycopersici* treatment did not change the shoot weights compared to the *F. oxysporum* f. sp. *lycopersici* treatment. There was a low positive correlation between shoot weight and AM colonisation levels (*P* < 0.01, *R* ^*2*^ = 0.040); however, shoot weight was negatively correlated with disease severity (*P* < 0.0001, *R* ^*2*^ = 0.200).

## Discussion

Mycorrhizal symbiosis with its aspects of biofertilization and bioprotection is of special interest in the context of sustainable agriculture. The present work assessed the impact of AMF in an intercropping system with the main focus on the performance of tomato (cv. Kremser Perle). As far as AM root colonisation of tomato is concerned, this was clearly affected by the intercropping plant partners. Tomato intercropped with leek showed a 20 % higher colonisation level than tomato intercropped with tomato. Fennel on the other hand decreased the colonisation level of tomato by 13 %. Leek had a similar colonisation level (55.11 %) as its intercropping partner tomato. However, the AM colonisation values of leek had a very high standard deviation, probably due to the rather small root system leading to a small amount of material for AM colonisation assessment. The well-established AM symbiosis in leek stimulated the colonisation of tomato, an effect that is concordant with data reported by Cavagnaro et al. (2004), and additionally, is in line with the frequent use of leek as nurse plants in experimental set-ups (Smith and Read 2008). However, a high AM colonisation level of the intercropping partner does not necessarily imply an increase in colonisation of the other one, as can be seen in the results with cucumber and fennel.

The small size of the rooting system of leek and as a consequence a faster colonisation of the root system and development of hyphal networks, which are apart from spores and root fragments, of high significance in AM root colonisation (Smith and Read 2008) might be the reason for the increased colonisation rates compared to intercrops like tomato, cucumber, basil and fennel. Furthermore, putative colonisation preferences could have more impact when larger root systems are available. The colonisation preference observed for cucumber of the AMF used in the present work is consistent with the findings of Kubota and Hyakumachi (2004), who tested cucumber as well as tomato in soils of different vegetation sites with native AMF species and found a clear colonisation preference of AMF for cucumber; however this is not in an intercropping setting with different AMF. The importance of intercropping or individual settings can be seen in the work of Cavagnaro et al. (2004), who reported that mycorrhizal responsiveness of the tomato variety 76R to *Glomus coronatum* depended on the presence or absence of a mycorrhiza-defective tomato mutant derived from cv. 76R (i.e. a setting with interspecific competition). It therefore appears probable that the outcome of an AMF–plant interaction in intercropping settings cannot be predicted from individual settings. The significance of the intercropping partner for AM colonisation levels is clearly shown in the present study on tomato.

The investigation of the influence of AMF and/or *F. oxysporum* f. sp. *lycopersici* on growth of intercropped tomato showed that the intercropping partners of tomato impacted on root and shoot weight. Most striking was the reduction of 50 and 40 %, respectively, in the root and shoot weights of one tomato plant/pot in the TT control treatment compared to the other tomato plant. AMF did not change the effects of the intercropping partner on these growth parameters compared to the control treatment. Root and shoot weight increases were AMF-dependent and not intercropping partner-dependent. Schroeder-Moreno and Janos (2008) found that AMF had negative effects on the root as well as on the shoot weights of chilli, maize and zucchini grown in intraspecific density settings. Negative effects on tomato root or shoot weights due to AMF could not be observed in the present work. However, lowest density here was two and not three plants, as in the work of Schroeder-Moreno and Janos (2008). With regards to interspecific competition, van der Heijden et al. (2003) clearly showed that the AMF species can influence the outcome of a competitive situation between plants. Working with *Brachypodium pinnatum* and *Prunella vulgaris*, these authors observed that the way in which the two plants coexisted depended on the *Glomus* isolate inoculated. In the present work, six different *Glomus* species were inoculated together; it would be interesting to test different AMF inocula separately in the same intercropping setting to find out more about such dynamics.

When assessing the influence of AMF inoculation in intercropping systems on *F. oxysporum* f. sp. *lycopersici* disease severity in tomato, a bioprotective effect was observed. This bioprotective effect resulted in a reduced disease severity in AM plants of the T_1, TL and TB treatments. Looking on T_1, TL and TF, AMF + *F. oxysporum* f. sp. *lycopersici*-treated plants produced more shoot biomass compared to the *F. oxysporum* f. sp. *lycopersici*-treated plants. Thus, mycorrhization enhances the tolerance of the tomato plants to the pathogen. Positive effects of AMF, expressed as an increase in biomass compared to the control treatment, could be observed for root as well as shoot weights. For roots, these effects could be observed for the AMF treatment in T_1 of TL and TF, whilst for shoots, the positive effects on biomass could be observed for AMF as well as AMF + *F. oxysporum* f. sp. *lycopersici* treatments in T_1 of TT, TL and TF combinations. *F. oxysporum* f. sp. *lycopersici* clearly reduced and AMF clearly increased shoot biomass for T_2 and TL. Consequently, the positive effects of AMF co-inoculation with *F. oxysporum* f. sp. *lycopersici* on plant biomass are shown in shoot weights and not in reduced disease incidence. Compensation by AMF for the negative effects of *F. oxysporum* f. sp. *lycopersici* on plant biomass could particularly be reached with leek as intercropping partner. Thus, a well-chosen intercropping partner like leek and basil can allow expression of a bioprotective effect of AMF, even when the symbiosis is not established before pathogen inoculation. A different experimental set-up, like in Dehne and Schönbeck (1979), where AMF were applied 6 and 9 weeks, respectively, before *F. oxysporum* f. sp. *lycopersici* inoculation might have led to more positive effects. However, a simultaneous inoculation in an intercropping setting appears to be more comparable to natural conditions where concurrent activity of AMF and *F. oxysporum* f. sp. *lycopersici* will not be uncommon.

When tomato was intercropped with tomato, one intercropping partner turned to be the ‘stronger’ and the other the ‘weaker’ one, which also clearly affected the disease incidence and disease severity. Thus, keeping tomato in a competitive situation with itself has adverse effects on the biomass of one of the intercropping partners. The ‘weaker’ partner also showed significantly higher *F. oxysporum* f. sp. *lycopersici* disease severity and no positive effects of AMF inoculation. Therefore, we conclude that the positive effects of AMF on disease severity are limited in competitive situations, and that the intercropping partner affects the positive effects of AMF on tomato plants with regard to *F. oxysporum* f. sp. *lycopersici* disease severity. Results indicate that leek and basil are candidates for further intercropping set-ups, where further questions like fruit yield and fruit quality of tomatoes should be assessed and also the impact of pre-colonisation by AMF of intercropping partners could be investigated.

To summarise, tomato intercropped with different species had no effect on *F. oxysporum* f. sp. *lycopersici* disease incidence or disease severity indicating no allelopathic suppression. However, tomato intercropped with tomato clearly showed negative effects on one plant/pot with regard to biomass and disease severity of *F. oxysporum* f. sp. *lycopersici*. Furthermore, crucial effects of the intercropping partner on AMF colonisation of tomato were found, which is of great interest in crop plant communities and the influences on each other. However, the outcome of the AMF effects on *F. oxysporum* f. sp. *lycopersici* disease severity and/or plant biomass did not depend on the degree of AM colonisation but more on the intercropping partner.
